# Optimization of *piggyBac* transposon-mediated gene transfer method in common marmoset embryos

**DOI:** 10.1371/journal.pone.0287065

**Published:** 2023-06-09

**Authors:** Nanami Kohri, Mitsuo Ota, Hikaru Kousaku, Eiko N. Minakawa, Kazuhiko Seki, Ikuo Tomioka

**Affiliations:** 1 Laboratory of Applied Reproductive Science, Faculty of Agriculture, Shinshu University, Nagano, Japan; 2 Department of Neurophysiology, National Institute of Neuroscience, National Center of Neurology and Psychiatry, Tokyo, Japan; Friedrich-Loeffler-Institute, GERMANY

## Abstract

Generating non-human primate models of human diseases is important for the development of therapeutic strategies especially for neurodegenerative diseases. The common marmoset has attracted attention as a new experimental animal model, and many transgenic marmosets have been produced using lentiviral vector-mediated transgenesis. However, lentiviral vectors have a size limitation of up to 8 kb in length for transgene applications. Therefore, the present study aimed to optimize a *piggyBac* transposon-mediated gene transfer method in which transgenes longer than 8 kb were injected into the perivitelline space of marmoset embryos, followed by electroporation. We constructed a long *piggyBac* vector carrying the gene responsible for Alzheimer’s disease. The optimal weight ratio of the *piggyBac* transgene vector to the *piggyBac* transposase mRNA was examined using mouse embryos. Transgene integration into the genome was confirmed in 70.7% of embryonic stem cells established from embryos injected with 1000 ng of transgene and transposase mRNA. Under these conditions, long transgenes were introduced into marmoset embryos. All embryos survived after transgene introduction treatment, and transgenes were detected in 70% of marmoset embryos. The transposon-mediated gene transfer method developed in this study can be applied to the genetic modification of non-human primates, as well as large animals.

## Introduction

Non-human primates are similar to humans in terms of genetics, physiology, and morphology, and the production of transgenic non-human primates can support modeling of various human diseases. Among non-human primates, the common marmoset (*Callithrix jacchus*) has a relatively short gestation period and prolific fecundity, contributing to the rapid establishment of transgenic model lines. Although transgenesis in mammals has been successfully developed in numerous studies using mice that are fertile and easy to handle, techniques for non-human primates have not progressed because of their rarity and difficulty in handling these animals. One of the few transgenesis techniques applied to non-human primates is the lentiviral vector-mediated gene transfer method, which is less invasive and allows for highly efficient gene transfer because of the injection into the perivitelline space of preimplantation embryos. Many transgenic marmosets have been produced using lentiviral vectors [1–3]. However, lentiviral vectors cannot introduce transgenes longer than 8 kb into the host genome [4], making it impossible to create transgenic marmosets with long pathogenic genes, long tissue-specific promoters, and advanced gene expression systems, such as controllable transgene expression systems. To generate diverse human disease model marmosets, transgenic techniques that introduce lengthy constructs into embryos should be developed.

Transposon-mediated gene transfer techniques have been applied to a wide range of organisms as a method that can introduce long genes. Transposons are mobile genetic factors that integrate their genes into the genomes of host cells [5]. Transposon elements contain terminal inverted repeats that interact with transposase, which cuts the transposon gene from the element and integrates the excised gene into a new location in the target DNA. The major species of transposons used for producing transgenic mammals are *piggyBac*, *Sleeping Beauty* [6, 7], and *Tol2* [8–11]. Among them, the *piggyBac* transposon discovered from the cabbage looper moth (*Trichoplusia ni*) and baculoviruses [12–14] can mobilize 207 kb DNA fragments in human cells and mice [15]. Thus, transposon-mediated gene transfer techniques are expected to be powerful tools for creating human disease models on the common marmoset.

Difficulties in non-human primate transgenesis include the rarity of the animals and the complexity of collecting fertilized embryos. In general, one-cell stage embryos are suitable for transgenesis, but surgical procedures are required for their collection in non-human primates. This necessitates considerable manpower and substantial wear and tear of the animals. However, for the collection of four- to eight-cell stage embryos, minimally invasive and nonsurgical uterine perfusion methods can be applied [1–3, 16], allowing the same animals to be used continuously for experiments over a long period of time. In this study, we optimized a transposon-mediated gene transfer method in which transgenes longer than 8 kb were injected into the perivitelline space of four- to eight-cell stage embryos, followed by electroporation. The transgenesis technique developed in this study may contribute not only to the highly efficient creation of various disease models on the common marmoset, but can also be applied to other non-human primates and large animals.

## Materials and methods

### Ethical approval

All animal experiments using mice were conducted in accordance with the institutional guidelines established by Shinshu University for the care and use of laboratory animals and were approved by the Ethics Committee for Animal Research of Shinshu University, Japan. All animal experiments using common marmosets were approved by the Ethics Committee for Primate Research of the National Center of Neurology and Psychiatry, Japan.

### Animals

The present study was conducted in compliance with the animal research: reporting of in vivo experiments (ARRIVE) guidelines. All experiments using mice were conducted in accordance with the institutional guidelines established by Shinshu University for the care and use of laboratory animals and were approved by the Ethics Committee for Animal Research of Shinshu University, Japan. ICR mice were purchased from Japan SLC (Shizuoka, Japan) and housed in a temperature-controlled room under a 12 h/12 h light-dark cycle. All animal experiments using common marmosets were approved by the Ethics Committee for Primate Research of the National Center of Neurology and Psychiatry in Japan and were conducted in accordance with institutional guidelines and the National Research Council’s Guide for Care and Use of Laboratory Animals. All animals were maintained on a 12-hour light/dark cycle (7:00 AM/7:00 PM) and were individually housed in cages with wire mesh floors maintained at 27–29°C with 45–55% humidity. Wooden perches for locomotion and a platform for a bed were placed in each cage for environmental enrichment. All were fed commercial monkey food CMS-1M (FEED ONE CO., LTD., Japan) twice daily. Animals were provided with tap water ad libitum from feed valves. Daily care was provided by the same staff, and the veterinary staff took care of the animals in case of any health problems. The veterinary staff also examined the condition of the animals before and after the study. For the euthanasia procedure, marmosets were sedated with an injection of ketamine hydrochloride (30 mg/kg, i.m.) followed by an injection of sodium pentobarbital (40 mg/kg i.p.; Somnopentyl, Kyoritsu Seiyaku, Tokyo, Japan). The abdomen was then opened and blood was released from the abdominal aorta.

### Mouse and common marmoset embryo collection

Female ICR mice were superovulated by injection of 10 IU of pregnant mare serum gonadotropin (ASKA Animal Health, Tokyo, Japan) and 10 IU of human chorionic gonadotropin (ASKA Animal Health), administered 48 h apart. The two-cell stage embryos were collected in M2 medium (Sigma-Aldrich, St. Louis, MO, USA) from the oviducts of intercrossed females at E1.5. The embryos were transferred to M16 medium (Sigma-Aldrich) and cultured overnight at 37°C in a humidified atmosphere of 5% CO_2_ and 5% O_2_ until four- to eight-cell stage. Marmoset oocyte collection and intracytoplasmic sperm injection were performed as described previously [2, 17]. Briefly, ovaries were obtained from female marmosets euthanized in another experiment, and immature oocytes were collected by puncturing the follicles in POM (FUJIFILM, Osaka, Japan) using a sterile 26 G needle on a syringe. After three washes, approximately 10–20 immature oocytes were cultured in 100 μL droplets of POM supplemented with 5% FBS and 100 mIU/mL follicle-stimulating hormone. After 36 h of in vitro maturation culture, mature oocytes were fertilized with sperm in M2 medium (Sigma-Aldrich) by intracytoplasmic sperm injection using a micromanipulator (Narishige, Tokyo, Japan) with a piezo drive system (Primetech). The embryos were subsequently cultured in TYH medium for 1 day and in sequential blast medium (ORIGIO, Målov, Denmark) for 2 days. Embryos at the four- to eight-cell stage were vitrified with Vitrification Media (Kitazato BioPharma, Shizuoka, Japan) according to the manufacturer’s instructions. Briefly, embryos were equilibrated in ES solution for 10–15 min and then transferred to VS solution for 1–1.5 min at room temperature. The embryos were loaded onto Cryotop (Kitazato BioPharma) with a minimal amount of VS solution and quickly stored in liquid nitrogen until use. The vitrified embryos were thawed using Thawing Media (Kitazato BioPharma) according to the manufacturer’s instructions. Briefly, the embryos were quickly transferred to TS solution for 1 min at 37.0°C. The embryos were then transferred to the DS solution and incubated for 3 min at room temperature, followed by incubation in WS solution for 5 min at room temperature. Embryos were cultured in sequential blast medium (ORIGIO, Målov, Denmark) for 3–6 h and subjected to the further experiments.

### Plasmid construction

**Fig 1:**
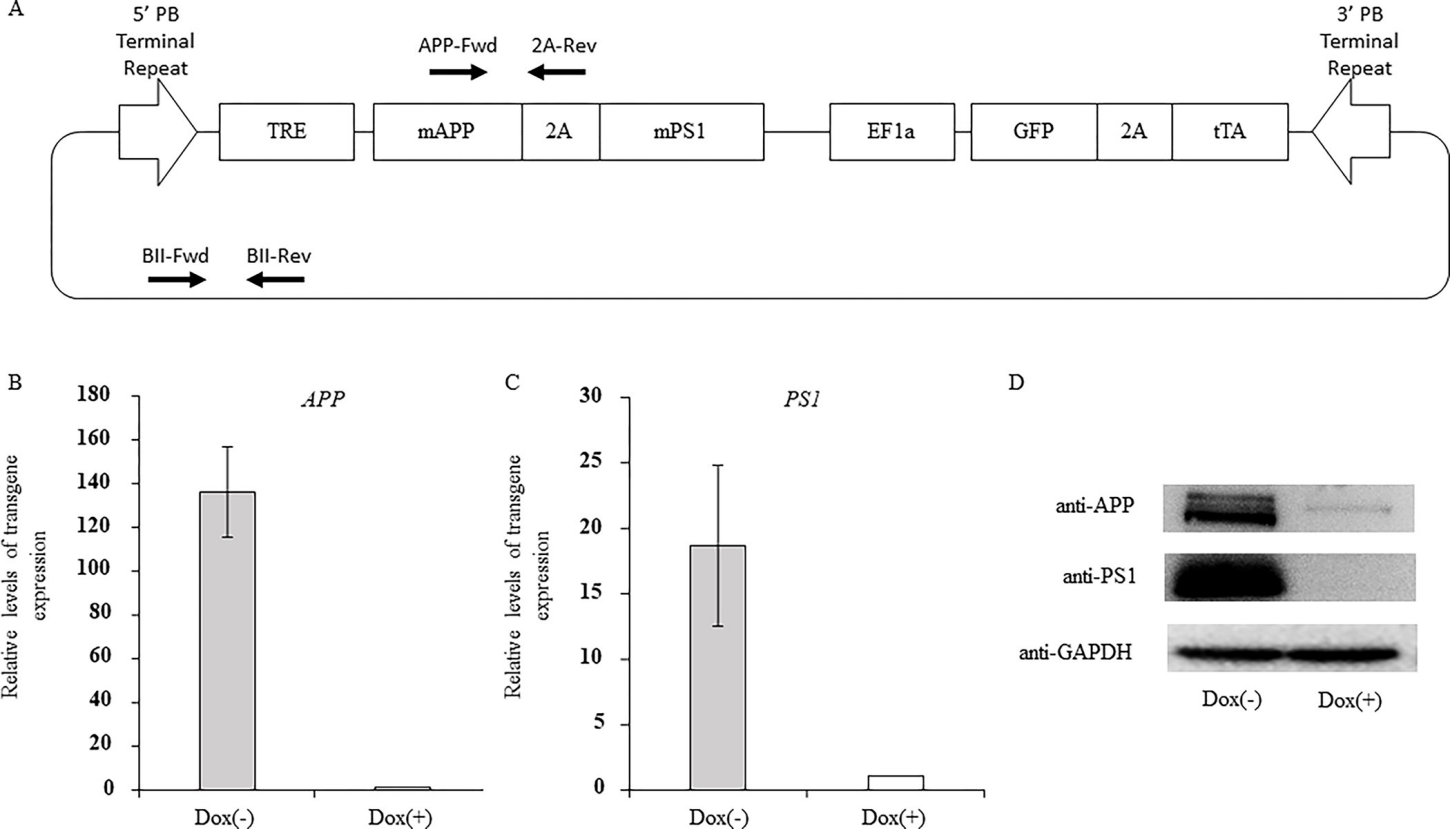
Construction of *piggyBac* vector carrying mutant APP and PS1 genes driven by the Tet-Off system. (**A**) The schematic representation of constructed vector. The mutant human APP and PS1 were linked by the 2A sequence, and the expression was controlled by the tetracycline-responsive element (TRE) promoter. The green fluorescent protein (GFP) and tTA were linked by the 2A sequence and expression was controlled by the elongation factor 1-alpha (EF1a) promoter. The arrows indicate primers to detect transgene. (**B and C**) The expression levels of mutant human *APP* (B) and *PS1* (C) mRNA controlled by Tet-Off system. The expression levels of mutant human *APP* and *PS1* in 293FT cells with or without doxycycline treatment are depicted. (**D**) Western blotting analysis for mutant APP (top panel), mutant PS1 (middle panel), and glyceraldehyde 3-phosphate dehydrogenase (GAPDH) (bottom panel) in 293FT cells with or without doxycycline treatment.

All transgene constructions were performed using the In-Fusion® HD Cloning Kit (Takara Bio, Shiga, Japan) according to the manufacturer’s instructions. To construct PB-TRE-APP-2A-PS1-EF1α-GFP-2A-tTA (Fig 1A), the full-length human mutant amyloid precursor protein (APP) (Swedish mutation K670N/M671L, Florida mutation I716V, and London mutation V717I) and presenilin-1 (PS1; M146L and L286V) [18] were linked using a self-cleaving T2A peptide (2A) sequence, and the resulting constructs were inserted downstream of the TRE region of a self-inactivating lentiviral vector (CSIV-RfA-TRE-EF-KT; RIKEN, Tsukuba, Japan). A marker gene encoding the green fluorescent protein (GFP) and tTA sequence was linked by a 2A sequence, and the resultant constructs were inserted downstream of the elongation factor 1-alpha (EF1a) promoter of a self-inactivating lentiviral vector (RIKEN). Finally, the backbone of the lentiviral vector was exchanged with that of the PB713B-1 vector (System Biosciences, CA, USA), and the length of the transgene inserted into the genome was approximately 9 kb. The transposon vector and Super PiggyBac Transposase (PBtp) Expression Vector (PB210PA-1; System Biosciences) were co-transfected to introduce transgenes into cells or embryos.

### Transfections with donor plasmids into 293FT cells

293FT cells at a density of 1.5 × 10^6^ cells per well were plated onto 60 mm culture dishes coated with 0.01 mg/mL poly-L-ornithine (FUJIFILM) per dish. The cells were cultured overnight in Dulbecco’s modified Eagle’s medium (DMEM; FUJIFILM) supplemented with 10% (v/v) fetal bovine serum (FBS; FUJIFILM) and 1% (v/v) penicillin-streptomycin solution (Nacalai Tesque, Kyoto, Japan). Vectors were transfected using ScreenFect^TM^ A (FUJIFILM), according to the manufacturer’s instructions. Briefly, 293FT cells were cultured in Opti-MEM (Thermo Fisher Scientific, Inc., Waltham, MA, USA) supplemented with ScreenFect^TM^ A and donor plasmids for 6 h. After transfection, the cells were treated with 2 μg/mL doxycycline in DMEM supplemented with 10% (v/v) FBS and 1% (v/v) penicillin-streptomycin solution overnight.

### RNA extraction, reverse transcription, and quantitative polymerase chain reaction (qPCR) analysis

Total RNA from 293FT cells was extracted using NucleoSpin® RNA Plus (Takara Bio), according to the manufacturer’s instructions. First-strand cDNA was synthesized from the extracted RNA using the ReverTra Ace qPCR RT Master Mix with gDNA Remover (FSQ-301, Toyobo, Osaka, Japan). The primer sets used for the qPCR analysis are listed in S1 Table. Plasmids or cDNA were subjected to qPCR. Thermal cycling conditions for plasmids consisted of one cycle at 95°C for 30 s, followed by 45 cycles at 95°C for 5 s and 60°C for 30 s. On the contrary, thermal cycling conditions for cDNA consisted of one cycle at 95°C for 30 s, followed by 35 cycles at 95°C for 10 s, 60°C for 15 s, and 72°C for 15 s. Real-time qPCR was performed using Thunderbird NEXT (Toyobo, Tokyo, Japan) for plasmids and TB Green® Premix Ex Taq™ II (Tli RNaseH Plus, Takara Bio) for cDNA, according to the manufacturer’s instructions. The results of qPCR using cDNA were normalized to β-actin expression, and the relative transgene expression levels were analyzed using the ΔΔCt method.

### Western blotting analysis

Proteins from 293FT cells were extracted using the EzRIPA Lysis Kit (ATTO, Tokyo, Japan) according to the manufacturer’s instructions. The proteins were treated at 70°C for 15 min before electrophoresis. For western blotting analysis, 70 μg of protein per lane was electrophoresed on a 10% (w/v) polyacrylamide gel and transferred to a polyvinylidene difluoride membrane (FUJIFILM). The membrane was blocked with EzBlock Chemi (ATTO) for 1 h and incubated overnight with the following antibodies: anti-APP (LN27, mouse monoclonal, 1:1000; BioLegend, San Diego, CA, USA), anti-PS1 (NT1, mouse monoclonal, 1:1000; BioLegend), and anti-GAPDH (5A12, mouse monoclonal, 1:10000; FUJIFILM). Subsequently, the membrane was incubated for 50 min at room temperature with horseradish peroxidase-conjugated goat anti-mouse IgG (Poly4053, polyclonal, 1:5000, BioLegend). Images of the protein bands were obtained using a LuminoGraph II (ATTO).

### In vitro transcription

The DNA fragments were constructed using the Super PiggyBac transposase expression vector (PB210PA-1, System Biosciences) by PCR amplification using specific primers (5’-GGCAGGTAATACGACTCACTATAGGGAGACGCATGGGCTCTAGCCTGGA-3’, 5’-GGCGGATCCTCAGAAACAGCTCTG-3’). PCR amplification was performed using KOD One® PCR Master Mix (Toyobo). Thermal cycling conditions consisted of one cycle at 98°C for 2 min, 30 cycles at 98°C for 10 s, 54°C for 30 s, and 72°C for 45 s, followed by 1 cycle at 72°C for 5 min. Amplified PCR products were electrophoresed at 100 V for 30 min on a 0.8% (w/v) agarose gel (Agarose S; NIPPON GENE, Tokyo, Japan) containing 0.0015% (v/v) SAFELOOK Pre-Green (FUJIFILM, Osaka, Japan). The PCR products were cut using a gel band cutter (FastGene, Tokyo, Japan). Gel extraction was performed using NucleoSpin Gel and PCR Clean-up (Takara Bio) according to the manufacturer’s instructions. Extracted and purified DNA fragments were used as templates for in vitro transcription. mRNA synthesis and poly (A) tailing were performed using the mMESSAGE mMACHINE® T7 Ultra Kit (Thermo Fisher Scientific Inc.) according to the manufacturer’s instructions. The synthesized mRNA was purified twice with NucleoSpin® RNA Clean-up (Takara Bio) and concentrated by centrifugation at 20,000 × *g* for 30 min at 4°C after adding 0.3 M sodium acetate (Nacalai Tesque) and 70% (v/v) ethanol (FUJIFILM). After incubation at −20°C for 30 min, the mRNA solution was centrifuged at 20,000 × g for 30 min at 4°C, followed by a wash with 99.5% (v/v) ethanol. Finally, the mRNA was dissolved in RNase-free water and stored at −60°C until use.

### Plasmid injection and electroporation

The plasmids and mRNA transposases for transgenesis were dissolved in OPTI-MEM (Thermo Fisher Scientific, Inc.). The embryos were transferred to M2 medium containing 0.1 M sucrose (Nacalai Tesque). The plasmids and mRNA were injected into the perivitelline space of embryos using an injection pipette with a spike (TPC, Målov, Denmark), followed by electroporation of the embryos in OPTI-MEM (S2 Table) using a Super Electroporator NEPA21 Type II (NEPA GENE, Chiba, Japan) and platinum plate electrodes on a glass slide (1 mm gap, L 15 mm× W 1 mm × H 1.5 mm). The embryos were incubated in M2 medium for 10 min at room temperature. For common marmosets, embryos were cultured in sequential blast medium for 7–14 days. After cultivation, the embryos were used for subsequent experiments.

### Establishment of embryonic stem cell (ESC) lines in mice

After cultivation of injected and electroporated embryos for 2 days, the zona pellucida of the blastocysts was removed using acidic Tyrode’s solution (pH 2.5, Sigma-Aldrich), followed by washing the blastocysts twice in M2 medium. Subsequently, the blastocysts were plated on 5 μg/mL mitomycin-treated mouse embryonic fibroblasts (MEFs) and cultured with D-MEM/Ham’s F-12 (FUJIFILM) supplemented with 10% (v/v) knockout serum replacement (Thermo Fisher Scientific, Inc.), 1% (v/v) GlutaMAX (Thermo Fisher Scientific, Inc.), 1% (v/v) MEM non-essential amino acid solution (FUJIFILM), 1% (v/v) 2-mercaptoethanol (FUJIFILM), and 1% (v/v) penicillin-streptomycin solution. After 2 weeks, the ESC-like colonies were dissociated with 0.25% (v/v) Trypsin-1 mM EDTA·4Na solution (FUJIFILM) and re-plated on MEFs. After 7 days, the ESC lines were subjected to DNA extraction.

### DNA extraction

The zona pellucida-removed preimplantation embryos were washed with phosphate-buffered saline solution, followed by transfer into 10 μL of distilled water per embryo, and incubated at 98°C for 10 min. DNA was isolated from mouse ESC lines using NucleoSpin® DNA RapidLyse (Takara Bio), according to the manufacturer’s instructions. Extracted DNA was stored at −30°C until use.

### PCR analysis

Primer sets used in this study are listed in S1 Table. PCR amplification was performed with Tks Gflex™ DNA Polymerase Low DNA (Takara Bio) and 0.3 μM each of the forward and reverse primers. Thermal cycling conditions consisted of one cycle at 94°C for 1 min, followed by 35 or 40 cycles at 98°C for 10 s, 60°C for 10 s, and 68°C for 24 s. Amplified PCR products were electrophoresed at 100 V for 30 min on a 1.5% (w/v) agarose gel (Agarose S; NIPPON GENE, Tokyo, Japan) containing 0.0015% (v/v) SAFELOOK Pre-Green (FUJIFILM).

### Statical analysis

Numeric differences in transgene integration rates were compared using the chi-square test. Differences were considered significant at p < 0.05.

## Results

### Construction of *piggyBac* vector carrying mutant APP and PS1 controlled by Tet-Off system

To confirm the expression of mutant APP and PS1, PB-APP-PS1 vector (Fig 1A) was transfected to 293FT cells, and the cells were cultured with or without doxycycline. Real-time PCR analysis revealed that mutant APP and PS1 mRNA expression levels decreased by 135.8- and 18.7-fold, respectively, after doxycycline treatment (Fig 1B and 1C). Western blotting analysis also showed that mutant APP and PS1 proteins dramatically decreased after doxycycline treatment (Fig 1D), indicating that the mutant APP and PS1 transgenes were accurately controlled by the Tet-Off system.

### Optimization of transposon-mediated gene transfer conditions in mice

**Fig 2:**
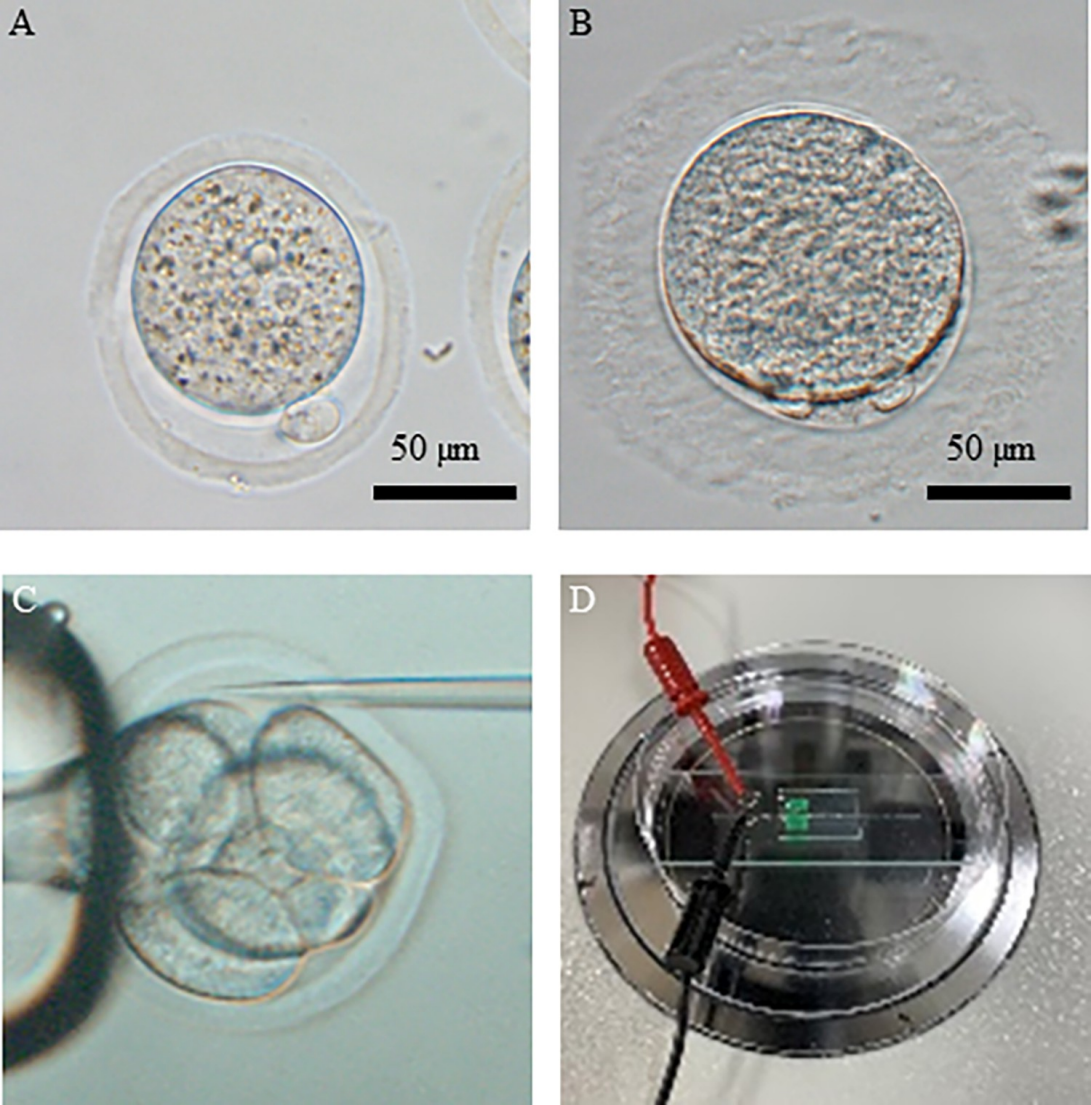
Transgene introduction into mouse and common marmoset embryos. (**A and B**) Mouse (A) and the common marmoset (B) embryos. (**C**) Transgene injection into the perivitelline space of embryos. (**D**) Platinum plate electrodes on slide glass.

Owing to the fact that the zonae pellucidae of common marmosets are extremely thick (Fig 2A) compared to those of mice (Fig 2B), PB-APP-PS1 and PBtp vectors were injected into the perivitelline space (Fig 2C), followed by electroporation (Fig 2D). To determine the optimal weight ratio of the PB-APP-PS1 vector to the PBtp vector, the experiment was conducted under the following four conditions: (i) the weight ratio of PB-APP-PS1 vector to DNA PBtp vector was 6.7:1; (ii–iv) three different weight ratios were considered for PB-APP-PS1 vector to mRNA PBtp vector, 2:1 (ii); 1:1 (iii); and 1:2 (iv). Compared to condition (i) with the DNA PBtp vector, more than 97.9% of embryos survived after transgene introduction under conditions (ii), (iii), and (iv) with mRNA PBtp vector (Table 1). The rates of development to the blastocyst stage were more than 93.3% in all four conditions, and the rates of ESC establishment ranged from 63.9% to 82.2%. To detect integrated transgenes, ESC lines were subjected to genomic PCR analysis. The highest gene transfer rate of 70.7% was observed under condition (iii) (Table 1).

**Table 1:** Optimization of transposon-mediated gene transfer conditions in mice.

			Number of embryos			Number of ESC lines		
No	Weight ratio (ng)		treated	survived after electroporation (%)	developed to blastocyst (%)	established (%)	examined for PCR analyses	positive for transgenes (%)
i	PB vector: DNA transposase	1000: 150	43	38 (88.4)	36 (94.7)	23 (63.9)	22	7 (31.8) ^ab^
ii	PB vector: mRNA transposase	1000: 500	47	46 (97.9)	43 (93.5)	34 (79.1)	34	18 (52.9) ^ac^
iii		1000: 1000	48	47 (97.9)	45 (95.7)	37 (82.2)	37	26 (70.7) ^c^
iv		1000: 2000	45	45 (100)	42 (93.3)	28 (66.7)	22	4 (18.1) ^b^

^a–c^ Different superscripts in each column indicate significant differences (p < 0.05).

### Embryo production by intracytoplasmic sperm injection, and long transgene introduction into common marmoset embryos via transgene injection into perivitelline space and electroporation

**Fig 3:**
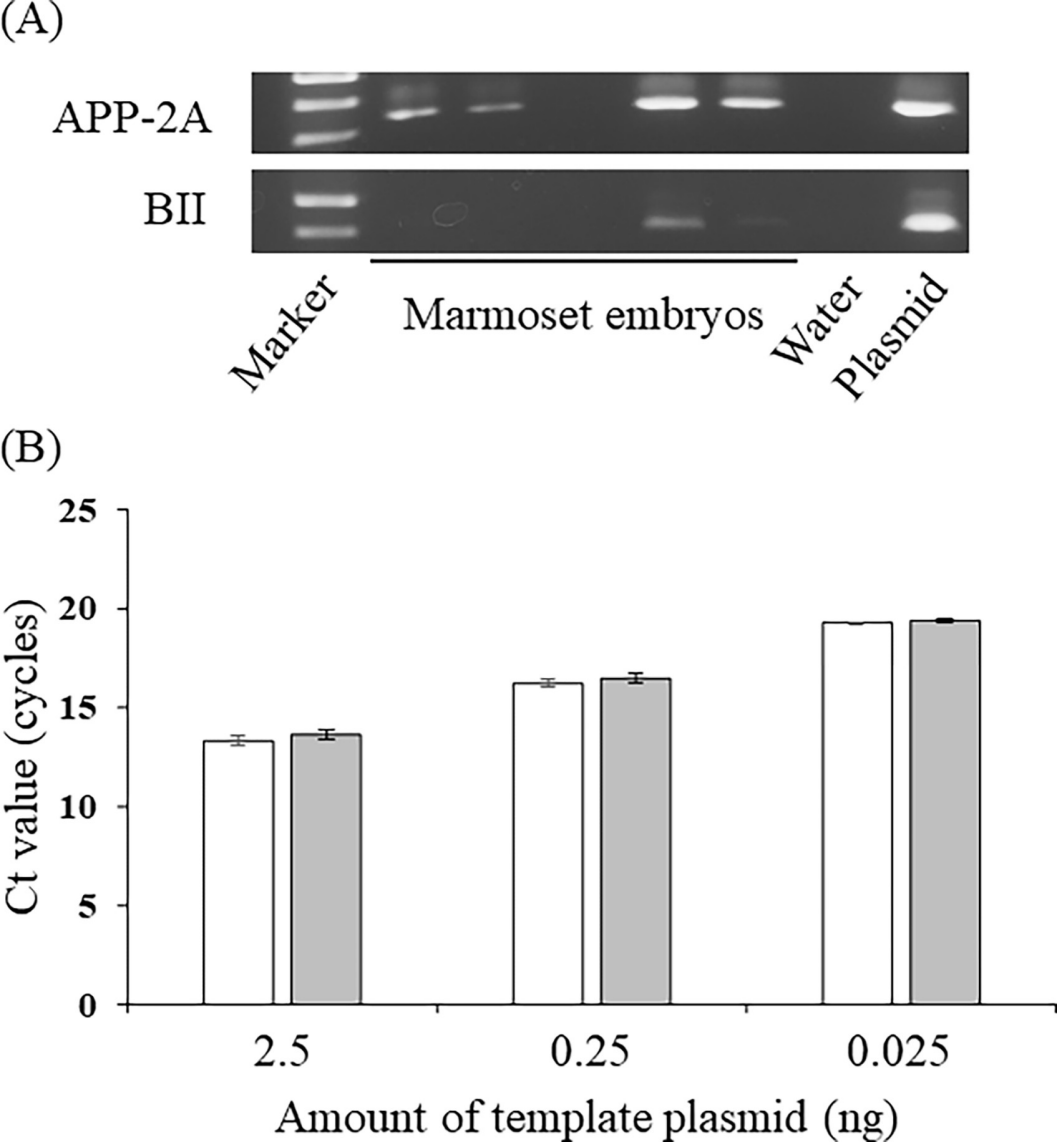
Transgene detection from the genomic DNA of common marmoset embryos. (**A**) PCR efficiency of the primer sets for BII (white) and APP-2A (light gray) analyzed by qPCR. Vertical and horizontal axis represent threshold cycle (Ct) values and the amount of template plasmid, respectively. (**B**) PCR analysis of genomic DNA from common marmoset embryos. The primer sets for APP-2A (upper panel) and BII (bottom panel) were used to detect genome integrated transgenes.

A total of 199 immature oocytes were collected from eight marmosets, and 112 (56.3%) of 199 oocytes matured in POM (Table 2). Almost all embryos survived intracytoplasmic sperm injection, with 49 embryos forming pronuclei and 23 embryos developing to the four- to eight-cell stage after 3 days of culture (Table 2). After freezing and thawing, 20 embryos survived and were subjected to subsequent experiments. Using the optimal conditions determined in the experiment with mouse embryos, the PB-APP-PS1 vector and mRNA PBtp vector at a 1:1 weight ratio were injected into the perivitelline space of the common marmoset embryos, followed by electroporation. Twenty embryos survived after transgene introduction (Table 3). After cultivation for 7–14 days, all embryos were subjected to genomic DNA extraction and PCR analysis. Genomic DNA PCR analysis showed that 17 embryos were positive for APP-2A, the insertion site of the transgene, and three of them were positive for BII, the backbone site of the transgene (Fig 3A). As a result, 14 out of 20 (70%) embryos were APP-2A positive and BII negative, which does not fully demonstrate but suggests successful transgene integration into the genome of marmoset embryos (Table 3). Meanwhile, qPCR was performed to determine whether the primer sets for APP-2A and BII had the same amplification efficiency. The results of qPCR analysis with 2.5, 0.25, and 0.025 ng of template plasmid showed that the amplification efficiencies of the APP-2A and BII primer sets were comparable (Fig 3B), showing that only one primer was not preferentially detected.

**Table 2:** Embryo production by intracytoplasmic sperm injection in common marmoset.

Number of animals	Number of oocytes			Number of embryos		
	collected	matured (%) *	survived after ICSI (%) **	formed pronucleus (%) **	developed to 4 to 8-cell stage (%) **	cryopreserved
8	199	112 (56.3)	111 (99.1)	49 (43.8)	23 (20.5)	23

* Percentage per collected oocytes.

** Percentages per matured oocytes.

**Table 3:** Long transgene introduction into common marmoset embryos via transgene injection into perivitelline space and electroporation.

PB vector: mRNA transposase weight ratio (ng)	Number of embryos					
	treated	survived after electroporation (%) *	subjected to PCR	positive for APP-2A (%) *	positive for BII (%) *	positive for APP-2A and negative for BII (%) *
1000: 1000	20	20 (100)	20	17 (85.0)	3 (15.0)	14 (70.0)

* Percentages per treated embryos.

## Discussion

Non-human primates, including the common marmoset, have attracted attention as useful models for medicine, especially in neurodegenerative diseases. Considering the fact that the number of oocytes and embryos that can be collected from the common marmoset is limited, the establishment of a highly efficient gene transfer technique without damage or loss in non-human primates is essential. As one of the major transgenic techniques for preimplantation embryos, transgene microinjection into pronuclei in one-cell embryos has been used for generating transgenic animals [19]. However, microinjection techniques require sophisticated skills and the survival rates of embryos after pronuclear microinjection are very low [20]. The final transgenesis efficiency in offspring is around 0.1−5% per embryo transferred surrogate, and the application of microinjections with embryo damage or loss is difficult in non-human primates’ transgenesis. Transgenesis by electroporation has recently been used in many experiments to create genetically modified animals because it does not require specialized techniques and causes less damage to embryos than microinjection. In rats, a higher proportion of electroporation-treated embryos reached the two-cell stage when compared to the microinjection-treated embryos [21]. Cleavage and developmental rates of blastocysts in porcine and bovine embryos after electroporation have been reported to be comparable to those in non-treated controls [22, 23]. Furthermore, the gene transfer method using electroporation improved the birth rates of transgenic embryos in mice [24, 25]. Consistent with these reports, in this study, more than 97.9% of the mouse embryos and all the common marmoset embryos survived after transgene introduction using electroporation. Although the development of marmoset embryos was poor because they were produced by intracytoplasmic sperm injection and freeze-thawed at the four- to eight-cell stage, the rates of development of blastocyst in electroporation-treated mouse embryos were high, indicating that gene transfer by electroporation causes little damage to embryos.

Electroporation also has the advantage of introducing transgenes into all blastomeres of four- to eight-cell stage embryos. In non-human primates, collecting one-cell stage embryos is rather difficult because of the need for surgical laparotomy, but four- to eight-cell stage embryos can be collected using a nonsurgical uterine perfusion method [1–3, 16]. The non-surgical embryo collection method causes less damage to the animals and allows the same animals to be used repeatedly over a longer period of time, resulting in a reduction in the number of animals utilized. Although four- to eight-cell stage embryos are not suitable for transgene microinjection because of the difficulty involved in introducing transgenes into all blastomeres, electroporation can deliver transgenes into all blastomeres of these embryos. However, the zonae pellucidae of preimplantation embryos prevent plasmid vector translocation from the outside into the cytoplasm in the electroporation-mediated gene transfer technique, especially in marmoset embryos with extremely thick zonae pellucidae. Therefore, we developed a highly efficient and low-damage gene transfer method by combining plasmid vector microinjection into the perivitelline space and electroporation-mediated transgene delivery into the embryos.

To improve the efficiency of transgenesis, transposon techniques have been applied to generate transgenic mice, and high transgenic efficiencies have been achieved [7, 26, 27]. Recently, in vitro synthesized transposase mRNA has been used [27, 28] for an effective and rapid transgene integration into the host genome. Suzuki et al. (2015) reported that the transgene-positive rate in the blastocyst stage was highest when the transgene and transposase mRNA were introduced at a 1:1 weight ratio [28]. Our results were consistent with this (Table 1). Notably, more than 70% of mouse ESC lines and marmoset embryos were transgene-positive, indicating that the method we developed is a powerful tool for the efficient generation of transgenic animals. However, gPCR analysis of transgene introduced-marmoset embryos has not yet demonstrated definite transgene integration into the genome, so ES cells and transgenic animals need to be generated in the future to verify the transgene integration into the genome. Based on the common marmoset data, we achieved the integration of long transgenes into the host genome by transposon-mediated gene transfer, highly efficient transgene transfer by injecting plasmid vectors into the pericellular area, and damage-free transgene transfer into developed embryos by electroporation. The method established in this study is expected to be useful for the genetic modification of non-human primates, as well as large animals.

## Supporting information

S1 Table Primer sequences used in this study. (PDF)

S2 Table Electroporation condition for embryos. (PDF)

S1 Raw images (PDF)
