# Supplementary material for: Optimization of piggyBac transposon-mediated gene transfer method in common marmoset embryos
Source: PLoS One. 2023 Jun 9;18(6):e0287065. doi: 10.1371/journal.pone.0287065 (PMC10256193; doi:10.1371/journal.pone.0287065)
Supplement: S1 Table — (PDF) [file pone.0287065.s001.pdf]

S1 Table. Primer sequences used in this study.

Primer name	Primer sequence
APP-forward	AAGTGAAGATGGATGCAGAAT
APP-reverse	ACCAAGGTGATGATGACCA
2A-reverse	CTCCACGTCACCGCATGTT
BII-forwad	AATCGGAACCCTAAAGGGAG
BII-reverse	TCATCTGCTCCATCACGCTG
PS1-forward	TGCCATCATGATCAGTGTC
PS1-reverse	AACACTTCCCCCAAGTAAATG
ACTB-forward	GATGGTGGGCATGGGTCAGAA
ACTB-reverse	AGCCACACGCAGCTCGTTGT
