# Supplementary material for: Optimization of piggyBac transposon-mediated gene transfer method in common marmoset embryos
Source: PLoS One. 2023 Jun 9;18(6):e0287065. doi: 10.1371/journal.pone.0287065 (PMC10256193; doi:10.1371/journal.pone.0287065)
Supplement: S2 Table — (PDF) [file pone.0287065.s002.pdf]

S2 Table. Electroporation condition for embryos.

	Voltage (V)	Pulse Length (msec)	Pulse Interval (msec)	Number of Pulses	Polarity	Decay Rate (%)
P-pulse	30.0	0.1	97	1	+	10.0
T-pulse	10.0	1.0	97	5	±	40.0
