# Supplementary figures and images for: Optimization of piggyBac transposon-mediated gene transfer method in common marmoset embryos
Source: PLoS One. 2023 Jun 9;18(6):e0287065. doi: 10.1371/journal.pone.0287065 (PMC10256193; doi:10.1371/journal.pone.0287065)

Fig. 1 (D)

anti-APP

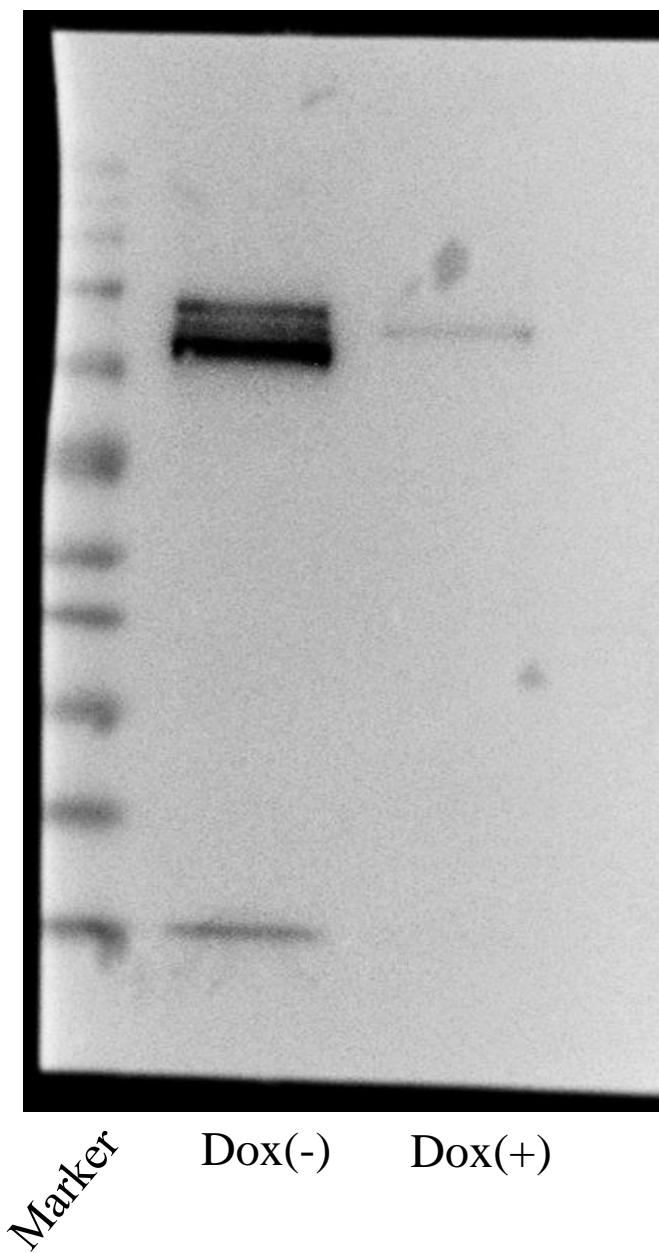

anti-PS1

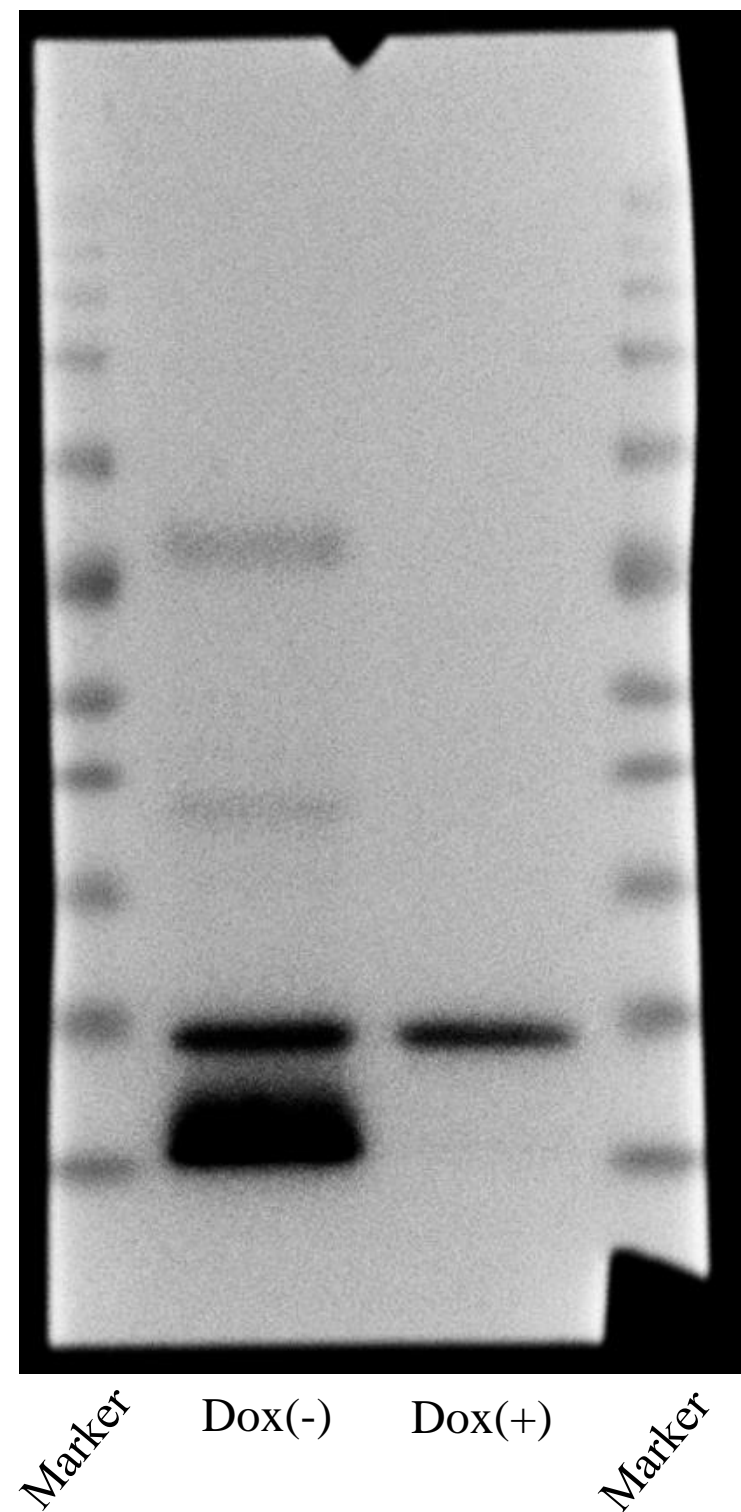

anti-GAPDH

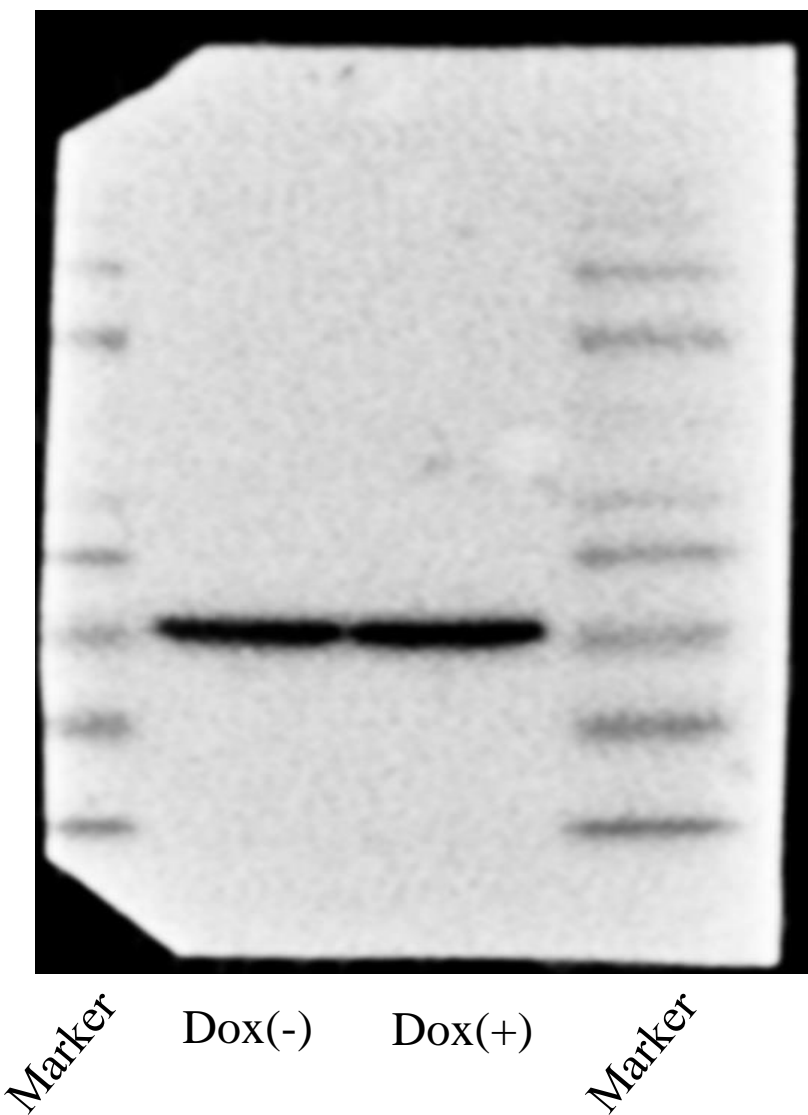

Fig. 3 (A)

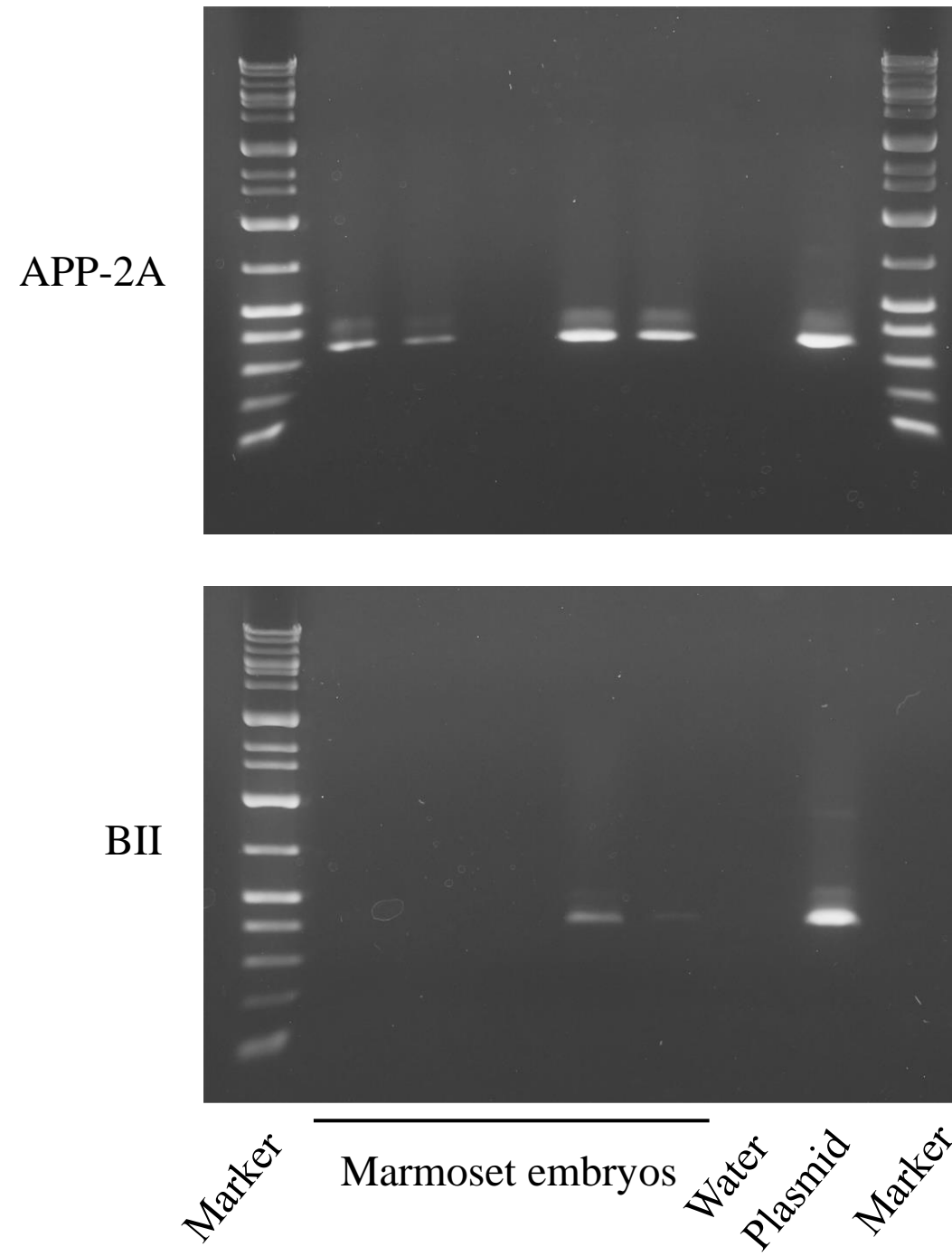

Supplement: S1 Raw images — (PDF) [file pone.0287065.s003.pdf]
